# Molecular Evolutionary Analysis of ABCB5: The Ancestral Gene Is a Full Transporter with Potentially Deleterious Single Nucleotide Polymorphisms

**DOI:** 10.1371/journal.pone.0016318

**Published:** 2011-01-27

**Authors:** Karobi Moitra, Mark Scally, Kate McGee, Germaine Lancaster, Bert Gold, Michael Dean

**Affiliations:** Laboratory of Experimental Immunology, Human Genetics Section, Cancer and Inflammation Program, National Cancer Institute at Frederick, Frederick, Maryland, United States of America; University of Cambridge, United Kingdom

## Abstract

**Background:**

ABCB5 is a member of the ABC protein superfamily, which includes the transporters ABCB1, ABCC1 and ABCG2 responsible for causing drug resistance in cancer patients and also several other transporters that have been linked to human disease. The ABCB5 full transporter (ABCB5.ts) is expressed in human testis and its functional significance is presently unknown. Another variant of this transporter, ABCB5 beta posses a “half-transporter-like” structure and is expressed in melanoma stem cells, normal melanocytes, and other types of pigment cells. ABCB5 beta has important clinical implications, as it may be involved with multidrug resistance in melanoma stem cells, allowing these stem cells to survive chemotherapeutic regimes.

**Methodology/Principal Findings:**

We constructed and examined in detail topological structures of the human ABCB5 protein and determined in-silico the cSNPs (coding single nucleotide polymorphisms) that may affect its function. Evolutionary analysis of ABCB5 indicated that ABCB5, ABCB1, ABCB4, and ABCB11 share a common ancestor, which began duplicating early in the evolutionary history of chordates. This suggests that ABCB5 has evolved as a full transporter throughout its evolutionary history.

**Conclusions/Significance:**

From our in-silco analysis of cSNPs we found that a large number of non-synonymous cSNPs map to important functional regions of the protein suggesting that these SNPs if present in human populations may play a role in diseases associated with ABCB5. From phylogenetic analyses, we have shown that ABCB5 evolved as a full transporter throughout its evolutionary history with an absence of any major shifts in selection between the various lineages suggesting that the function of ABCB5 has been maintained during mammalian evolution. This finding would suggest that ABCB5 beta may have evolved to play a specific role in human pigment cells and/or melanoma cells where it is predominantly expressed.

## Introduction

ABCB5 is a member of the ATP-binding cassette (ABC) superfamily of transporters that function in the ATP-dependent transport of structurally diverse molecules. A number of transporters in this family are implicated in multidrug resistance and are recognized causes for the failure of cancer chemotherapy [1], [2], [3].

These transporters represent the largest family of transmembrane proteins and are classified into seven families (A–G) in humans, based on the sequence and organization of the nucleotide-binding domain [4]. The conserved nucleotide binding domains of these transporters drive transport, whereas the more variable transmembrane domains create the translocation pathway, providing safe passage for a diverse variety of substrates [5].

The human genome contains 48 ABC genes; 16 of these have known functions, while 14 have been associated with human disease states [6]. ABCB5 is a member of the B branch of the ABC transporter superfamily which contains 11 members and includes the multidrug transporter P-glycoprotein (ABCB1), along with the bile salt exporter pump ABCB11 and the phosphatidylcholine transporter ABCB4 [7]. A number of distinct forms of ABCB5 have been shown to be expressed in various tissue types, including, but not limited to, melanocytes, melanoma cells, testis, mammary tissue, and retinal pigmented epithelium, [8], [9], [10], [11]. ABCB5 has also been found to be expressed at the transcriptional level in a number of cancer subtypes, including malignant melanoma, breast cancer, colorectal cancer and hepatocellular carcinoma [9], [12], [13], [14], and also has been linked to leukemia [15]. More importantly, it has been shown that this transporter may be expressed in melanoma stem cells which posses the CD133^+^ phenotype where it might mediate doxorubicin resistance. ABCB5 is also indicated to be a metastatic melanoma progression marker, and it is suggested that this transporter has a role in the regulation of progenitor cell fusion [9], [16]. Four major forms of human ABCB5 have been documented: ABCB5.a (812 aa, also known as beta form), ABCB5.e (134aa), ABCB5.f (131 aa, ABCB5 alpha form) and ABCB5.ts (1257aa, testis-specific) [17]. Of these, ABCB5.ts is a putative full transporter expressed specifically in the human testis. ABCB5.a is a half-transporter form expressed in melanocytes and melanoma. ABCB5.f (alpha) is the smallest predicted protein form (15 kD) and has been hypothesized to have a regulatory rather than a transport role [11].

Among the substrates of this rather elusive transporter are doxorubicin and rhodamine 123 [8], [13], [14]. There is evidence in the literature that shows inhibition of ABCB5 either at the protein or mRNA level can inhibit doxorubicin transport and also sensitize melanoma and liver cancer stem cells to the drug [9], [14]. To date, however, no exhaustive studies have been carried out regarding the organization, topology, evolution, and genetic variations of this transporter, leaving a knowledge gap in the field regarding basic questions such as: Does ABCB5 function as a full transporter or by dimerization of half-transporter units? How is this transporter structurally organized? How did this transporter evolve and how far back in time can its ancestry be traced? And finally, could genetic variants of this transporter potentially affect its functional role?

We have tried to bridge this gap by carrying out exhaustive in silico phylogenetic and bioinformatics studies, which indicate that this transporter evolved as a full, rather than a half-transporter, which implies that it functions as a full transporter in certain human tissues or developmental stages. The sequence and topological analysis of the half-transporter in humans shows a unique predicted topology in which the beta form of the transporter has two NBDs (nucleotide binding domains) instead of one, and the first NBD lacks the Walker A region, indicating that this NBD may not be completely functional. A number of single nucleotide polymorphisms (SNPs; 879 in total from NCBI dbSNP) have also been detected in/near this gene. Ten of these are non-synonymous (amino-acid changing) cSNPs, which may affect function. Non-synonymous cSNPs were present in key regions of the gene, including TMDs (transmembrane domains), NBDs, extracellular loops, and the intracellular loops. SNPs in ABC transporters have been reported to play an important role in patients' response to medication and also to play a pivotal factor in risk for disease [18].

## Results

### Prediction of topological structure with HMMTOP of the full-length ABCB5 indicates that it contains 12 transmembrane alpha helices, while ABCB5 beta has 6

**Figure 1:**
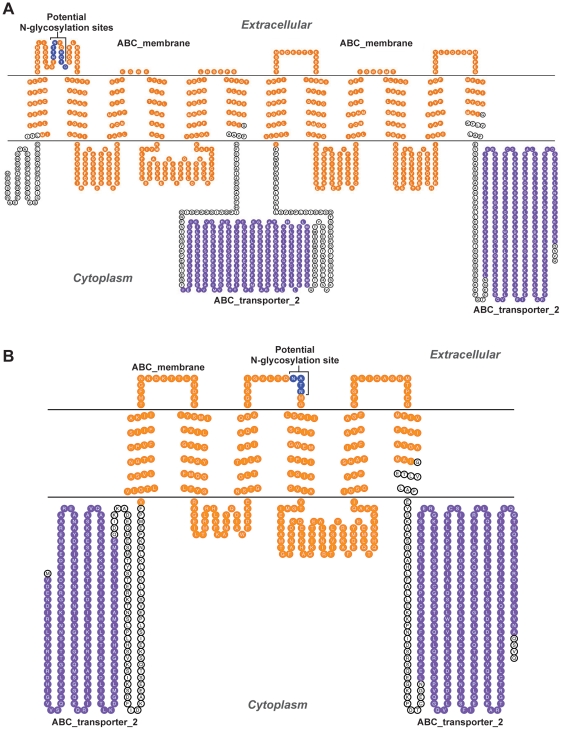
Predicted topological structures of ABCB5. **A. Topological structure of the ABCB5 full transporter.** The figure depicts 12 transmembrane alpha helices and 2 nucleotide-binding domains. The 2 ABC_membrane motifs, amino acids 48–339 and 692–968, are highlighted in orange and the 2 ABC_transporter_2 motifs, amino acids 386–622 and 1015–1253, are highlighted in purple. The 2 N-glycosylation sites, amino acids 85–88 and 91–94, which may be important for targeting of the transporter to the membrane, are highlighted in blue. The topological structure was determined using HMMTOP and displayed using the TOPO2 software. Functional motifs were determined using MOTIFSCAN. **B. Topology of ABCB5 beta.** Topology was determined with HMMTOP and displayed using TOPO2. Diagram depicts 6 transmembrane alpha helices and 2 nucleotide-binding domains. One ABC_membrane domain, amino acids 247–523, is shown in orange and the 2 ABC_transporter_2 domains, amino acids 2–177 and 570–808, are depicted in purple.

We determined the topology of the testis-specific full transporter and the beta form because they are most likely to have transporter functions. We constructed models using HMMTOP and displayed them using the program TOPO2. In the full length protein HMMTOP predicted the presence of the conventional 12 TMs with the 2 NBDs, which represents the accepted model for most full transporters (fig. 1A). For ABCB5 beta we found that the transporter had 6 predicted TMs, one complete NBD and one incomplete NBD. Both the NBD's are predicted to be located in the cytoplasm (fig. 1B).

Traditionally transporter topology has been determined using hydrophobicity plots which determine the locations of the hydrophobic and hydrophilic regions of the transporter (based on the properties of the amino acids) which correspond to the TM regions, extracellular/cytoplasmic regions respectively. Hydrophobicity plots for the full transporter were constructed with TMHMM which predicted 8 TM helices (data not shown). Hydrophobicity plots were also constructed for the beta form with TMHMM. The beta form was predicted to have 5 TM helices and the NBD was predicted to be on the extracellular surface, however when we aligned the ABCB5 beta sequence to human Pgp we found that the topology predicted that the transporter would have 6TM helices instead of 5 (data not shown). It is most likely that the structure predicted by HMMTOP is the closest prediction since it conforms to the basic structure of most ABC transporters, however, the likelihood that this transporter may have a unique structure cannot be completely ruled out.

### Functional motif analysis showed that ABC transporter motifs and potential membrane-targeting residues were present in ABCB5

The Motif Scan program was applied for predicting functional motifs within the human full-length ABCB5 amino acid sequence. The analysis revealed the presence of 2 ABC_transporter_2 motifs at amino acids 386–622 and 1015–1253; and 2 ABC_ membrane motifs at amino acids 48–339 and 692–968 (fig. 1A). The beta form showed 2 ABC_transporter_2 domain amino acids 2–177, 570–808, and one ABC_ membrane domain amino acids 247–523 (fig. 1B). Motif Scan was also used to detect functional domains. Several phosphorylation sites and glycosylation sites were present, which may be important for targeting of the protein to the membrane [19]. In the full transporter, 14 N-glycosylation sites were found, out of which 2 sites (aa 85–88, 91–94) may be important for targeting of the transporter to the membrane (fig. 1A). In the beta form, 6 N-glycosylation sites were found, out of which one site (amino acids 374–377) may be important for targeting of the transporter to the membrane (fig. 1B). A P-loop (Walker A) was detected at aa's 605–612 [AG]-x[4]-G-K-[ST]  =  (GSSGCGKST). The P-loop in ABC transporters is important in ATP binding and hydrolysis.

### Coiled-coil domain analysis of ABCB5 beta suggests that potential dimerization motifs are present at the N-terminal region

**Figure 2:**
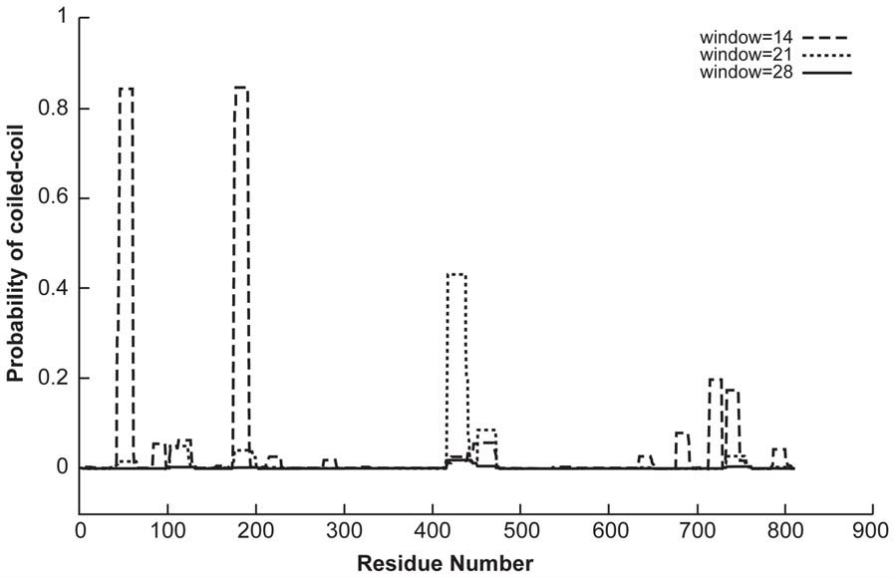
Coiled-coil regions of ABCB5 beta. Coiled-coil regions were calculated using the program COILS. These regions may indicate potential dimerization motifs in ABCB5 beta. Each alpha helix in a coiled coil is amphipathic, and the pattern of hydrophilic and hydrophobic amino acids repeats every 7 residues. Since the coiled-coil is a 7 residue heptad the sliding windows are set at multiples of 7, ie 14, 21 and 28 to enable the structure prediction algorithm to optimally detect the heptads.

The coiled–coil structure is a highly prevalent protein structural motif [20] and is thought to be present in approximately 5–10% of protein sequences [21]. Coiled–coils can have a stabilization function and are often involved in cellular signaling, protein interaction, and other significant cellular processes [20]. Two or more alpha-helices wound around each other in a symmetrical manner make up the basic “coiled–coil” structure [22]. Coiled-coils have been predicted to exist at the N-terminus of the goat half-transporter ABCG2 and the investigators have discussed the possibility that these domains may be involved in oligomerization functions [23]. A unique feature of the ABCB5 beta form is that it has 2 NBDs (first NBD lacking the Walker A) rather than the conventional 1 NBD, which is usually present in half-transporters. Due to this unusual configuration, we decided to investigate the presence of dimerization motifs in ABCB5—such as coiled–coil structures—using Marcoil, Coils and Psipred. Marcoil (v1.0) predicted coiled–coil domains in the N-terminal region of ABCB5 beta at a stringency of 1% in the three following regions: positions 46–58, 112–124, and 176–191. Coils (v1.0) predicted coiled–coil regions at or above 80% probability in 2 significant regions spanning amino acids 46–60 and 178–191 (fig. 2). These regions are similar to two of the coiled–coils predicted by Marcoil. Cross-checking these two regions with PSIPRED, predicted a coiled–coil domain extending from amino acids 177–193. Taken together, these results suggest that there is at least one coiled–coil motif in the N-terminal region of ABCB5. The coiled–coil motif may be used to control dimerization/oligomerization in different types of proteins. The coiled–coil is formed by the component helices coming together to bury their hydrophobic ends and form a super coil. Coiled–coil motifs are associated with different functions in proteins and thus may be very important to understand the functionality of the protein [19]. In the case of ABCB5 beta, they might be viewed as potential dimerization sites, which function to structurally bridge the potential half-transporter monomers to form the dimeric full transporter.

In ABCB5.ts 3 coiled coils in the N-terminus were also detected using the program Coils, spanning the amino acids 259-278, 490-505 and 621-636 giving rise to the speculation that possibly the smaller variant form of ABCB5 could be interacting with the coiled-coiled domains of the full transporter and may act in a dominant negative manner. Potential coiled-coil domains were also detected in the N-terminus of the B-family transporters ABCB1, ABCB4 and ABCB11(data not shown) which are the three transporters most closely related to ABCB5 suggesting that these domains are conserved in the B-family members closely related to ABCB5.

### Ten non-synonymous cSNPs are present in ABCB5 beta, while ABCB5.ts has 23

**Figure 3:**
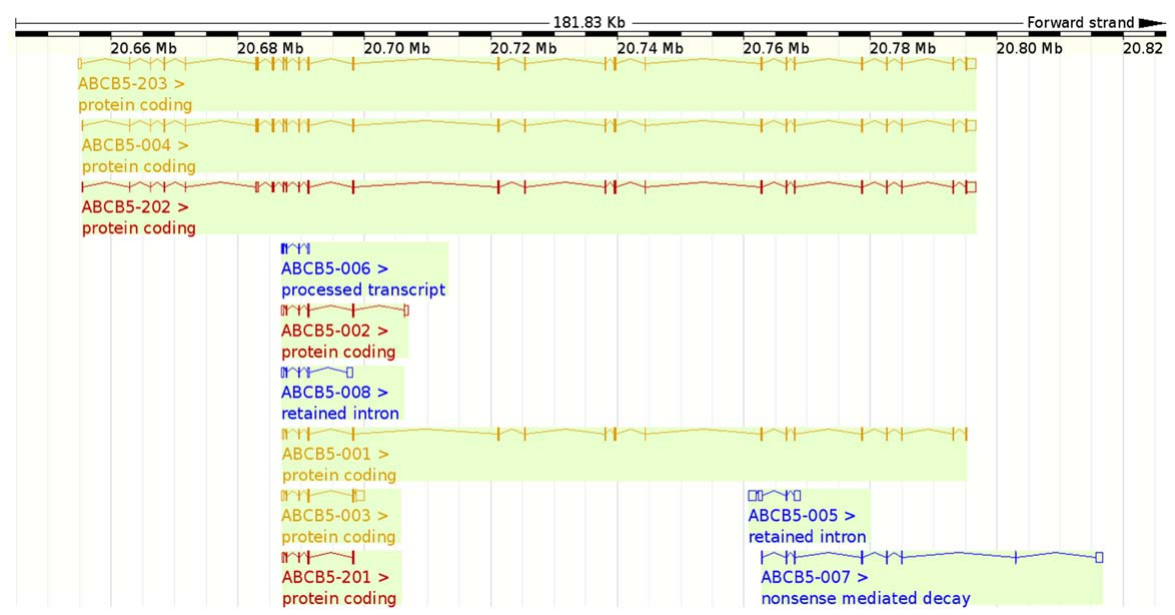
Genomic location (Chromosome 7p21) and multiple isoforms of the ABCB5. The figure depicts eleven ABCB5 splice variants (from *Ensemble*).

**Figure 4:**
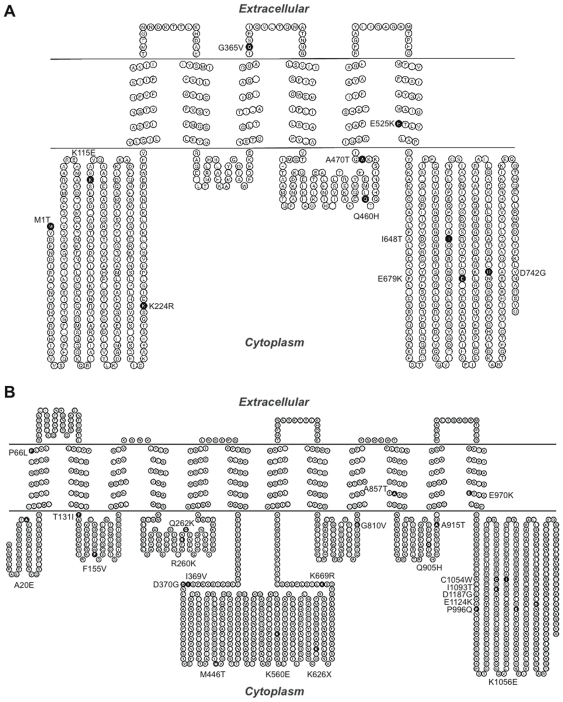
Non-synonymous cSNPs in ABCB5. **A. Topological model of predicted ABCB5 beta protein structure.** Diagram showing the locations of the 10 non-synonymous cSNPs. Black highlighted circles indicate ABCB5 non-synonymous cSNPs. TOPO2 was used to display annotated results. **B. Topological model of predicted ABCB5.ts (full length) protein structure** The model incorporates 23 ABCB5 non-synonymous cSNPs highlighted in black circles.

The *ABCB5* full transporter gene is located on chromosome 7p21, spans 28 exons and 141.8kb of genomic DNA. This gene encodes 11 different isoforms as depicted in ENSEMBL (fig. 3). The *ABCB5* beta gene is located on chromosome 7p21, spans 19 exons and 108 kb of genomic DNA [8]. SNPs in the transporter sequence were identified in the dbSNP database, and the cSNPs were mapped to the topological model of ABCB5 beta using the program TOPO2 (fig. 4A).

ABCB5 beta has 10 non-synonymous cSNPs documented by dbSNP. These are represented by ID numbers: rs34603556, rs2301641, rs13222448, rs35885925, rs17143304, rs6461515, rs60197951, rs59334881, and rs58795451. Using our knowledgebase of ABC transporters and the location of the SNPs on the protein, we compiled a list of the functional importance of these SNPs in regard to the protein (Table 1). From the table it can be seen that a number of the SNPs, rs2301641, rs13222448, and rs1143304, may affect signaling. rs58795451, rs59334881, and rs58795451 are located in the NBD; hence, they may be associated with aberrant ATP binding or hydrolysis. Only 1 SNP, rs6461515, is located within the substrates sites. Since this SNP may potentially affect substrate specificity, it may be functionally important in certain populations.

**Table 1:** Non-synonymous SNPs in the coding region of ABCB5 beta.

rs NUMBER	SNP	POSITION	SELECTION	POSSIBLE SIGNIFICANCE
rs34603556	1 M->T	Start	Neutral	Start site (ABCB5 beta)
rs2301641	115 K->E	Pot. NBD1	Purifying	Signalling
rs13222448	224 K->R	NBD1& TM1	Neutral	Signalling
rs62453384	365 G->V	Extracellular loop 2	Neutral	Signalling
rs35885925	460 Q->H	Cyt loop	Purifying	Potential Coupling helix
rs17143304	470 A->T	Cyt loop	Purifying	Signalling
rs6461515	525 E->K	TM 6	Purifying	Potential Substrate site
rs60197951	648 I->T	NBD2	Neutral	NBD Str/Funct./Sig.
rs59334881	679 E->K	NBD2	Purifying	NBD Str./Funct./Sig.
rs58795451	742 D->G	NBD2, D loop	Purifying	Communication between ATP sites

There are 23 non-synonymous cSNPs in the full transporter form of ABCB5.ts, and these are depicted in the protein (fig. 4B). The putative function of these residues is shown in Table 2. Of particular note is rs76179099, which would create a premature stop codon in the protein and may give rise to a truncated ABCB5 protein. The allele frequency for this SNP in a sample Yoruban (West African) population is 0.940 for the A allele (major allele) and 0.06 for the T allele (minor/SNP allele); data from dbSNP.

**Table 2:** Non-synonymous cSNPs (23) in the coding region of ABCB5 Full length (.ts).

rs NUMBER	SNP	POSITION	POSSIBLE SIGNIFICANCE
rs78309031	20 A->E	Cytoplasmic loop	Signaling
rs74552040	66 P->L	TM1	Potential substrate site
rs17143212	131 T->I	Cytoplasmic loop	Signaling
rs77409024	155 F->V	Cytoplasmic loop	Signaling
rs61741891	260 R->K	Cytoplasmic loop	Potential Coupling helix
rs2074000	262 Q->K	Cytoplasmic loop	Potential Coupling helix
rs58976125	369 I->V	NBD1	NBD Structure/Function/Signaling
rs61732039	370 D->G	NBD1	NBD Structure/Function/Signaling
rs34603556	446 M->T	NBD1	NBD Structure/Function/Signaling
rs2301641	560 K->E	NBD1	NBD Structure/Function/Signaling
rs76179099	626 K-> X	NBD1	Truncated protein
rs13222448	669 K->R	NBD1	NBD Structure/Function/Signaling
rs62453384	810 G->V	Cytoplasmic loop	Signaling
rs80123476	857 A->T	TM 10	Potential substrate site
rs35885925	905 Q->H	Cytoplasmic loop	Potential Coupling helix
rs17143304	915 A->T	Cytoplasmic loop	Potential Coupling helix
rs6461515	970 E->K	TM12	Potential substrate site
rs78765004	996 P->Q	NBD2	NBD Structure/Function/Signaling
rs80059838	1054 C->W	NBD2	NBD Structure/Function/Signaling
rs74333743	1056 K->E	NBD2	NBD Structure/Function/Signaling
rs60197951	1093 I->T	NBD2	NBD Structure/Function/Signaling
rs59334881	1124 E->K	NBD2	NBD Structure/Function/Signaling
rs58795451	1187 D->G	NBD2/D loop	Communication between ATP sites

### Functional analysis of cSNPs with Panther and SIFT (Sorting Intolerant from Tolerant) predicts that most of these SNPs might be deleterious to transporter function

The potential significance of these SNPs was determined using Panther, SIFT and our knowledge base of ABC transporters. The results are outlined in Table 3 and 4.

**Table 3:** In silico prediction of functional significance of ABCB5 beta non-synonymous cSNPs calculated using Panther.

Substitution	subPSEC score
M1T	−5.39815
K115E	−6.47709
K224R	−4.39767
G365V	−3.93172
Q460H	−4.45293
A470T	−4.2594
E525K	−4.30042
I648T	−6.31679
E679K	−4.85185
D742G	−8.71012

**Table 4:** In silico prediction of functional significance of ABCB.ts non-synonymous cSNPs calculated using Panther.

Substitution	subPSEC score
P66L	−5.10163
T131I	−3.1368
F155V	−7.05629
R260K	−2.69866
Q262K	−3.89135
I369V	−3.98202
D370G	−4.70914
M446T	−3.64169
K560E	−6.12726
K669R	−3.1384
G810V	−4.6663
A857T	−2.52579
Q905H	−4.03142
A915T	−3.86813
E970K	−2.81761
P996Q	4.62436
C1054W	−8.89455
I1093T	−4.64248
E1124K	−3.58989
D1187G	−6.60494

The Panther software calculates sub psec scores - substitution position-specific evolutionary conservation. It aligns the sequence of the transporter in various species, determines which of the positions are evolutionarily conserved among species, and assigns a sub psec score. A sub psec score of -3 or less means that the substitution has probable functional implications. From table 3 it can be seen that D742G has the lowest score (-8.01712), indicating a strong probability of functional impact. This agrees well with the fact that this residue, which is known as the “D” loop residue, has an important functional role in communicating signals from one NBD to another, suggesting that a substitution at this position may affect ATP hydrolysis by interfering with this signaling. Another important substitution would be M1T; since this is a start site substitution, any change in this residue would affect translation initiation. The SIFT algorithm also identified these substitutions as deleterious. Panther identified all the substitutions to be deleterious to the function of ABCB5 beta to some degree, whereas SIFT identified M1T (rs34603556), D742G (rs58795451), I648T (rs60197951), and K115E (rs2301641) to be deleterious. I648T is also located in the NBD, but not in a conserved motif, whereas K115E, which changes a positively charged amino acid to a negatively charged one, is located in a cytoplasmic loop and may be involved in signaling.

Among the ABCB5.ts SNPs (Table 4) C1054W present in NBD2 had the lowest sub-psec score (-8.89455), indicating that it was the most deleterious. This SNP would change the amino acid cysteine to a bulky tryptophan residue, which may affect NBD structure and function.

The D loop SNP D1187G had a score of -6.60494, indicating that this could also be deleterious to function (Note: Three of the residue changes, A20E, K1056E, and K626X, could not be calculated using Panther because the positions did not align to the hidden Markov model).

With the aid of SIFT analysis we found that a number of the substitutions were not tolerated including: F155V, R260K, Q262K, I369V, D370G, G810V, P996Q, C1054W, K1056E, E1124K and D1187G.

### Phylogenetic and Evolutionary Analysis

**Figure 5:**
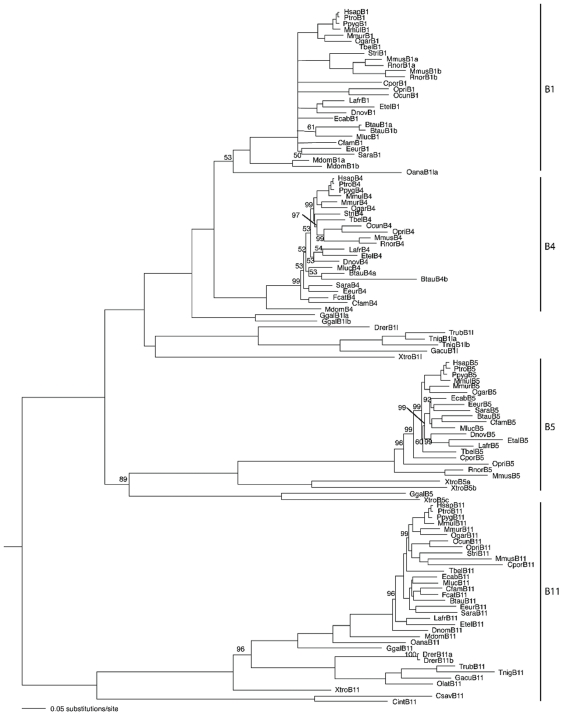
Phylogeny of the ABCB full transporters. The tree is a maximum likelihood phylogram, with Bayesian posterior probabilities (expressed as percentages). Support for each node is 100% unless otherwise indicated. Abbreviated species names are as follows; Hsap  =  *Homo sapiens*, Ptro  =  *Pan troglodytes*, Ppyg  =  *Pongo pygmaeus*, Mmul  =  *Macaca mulatta*, Mmur  =  *Microcebus murinus*, Ogar  =  *Otolemur garnettii*, Tbel  =  *Tupaia belangeri*, Stri  =  *Spermophilus tridecemlineatus*, Mmus  =  *Mus musculus*, Rnor  =  *Rattus norvegicus*, Cpor  =  *Cavia porcellus*, Opri  =  *Ochotona princeps*, Ocun  =  *Oryctolagus cuniculus*, Btau  =  *Bos taurus*, Ecab  =  *Equus caballus*, Cfam  =  *Canis familiaris*, Fcat  =  *Felis catus*, Mluc  =  *Myotis lucifugus*, Eeur  =  *Erinaceus europaeus*, Sara  =  *Sorex araneus*, Lafr  =  *Loxodonta africanus*, Etel  =  *Echinops telfairi*, Dnov  =  *Dasypus novemcinctus*, Mdom  =  *Monodelphis domestica*, Oana  =  *Ornithorhynchus anatinus*, Ggal  =  *Gallus gallus*, Xtro  =  *Xenopus tropicalis*, Drer  =  *Danio rerio*, Trub  =  *Takifugu rubripes*, Tnig  =  *Tetraodon nigroviridis*, Gacu  =  *Gasterosteus aculeatus*, Olat  =  *Oryzias latipes*, Csar  =  *Ciona savignyi*, Cint  =  *Ciona intestinalis*. Letters after the species name refer to the gene sequence, with an additional “l” indicating that the gene is not a direct ortholog, e.g., XtroB1l is a B1 “like” gene.

**Figure 6:**
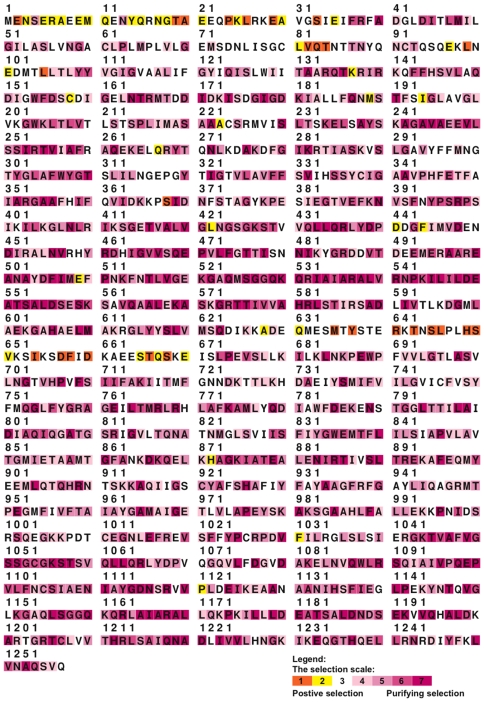
The level of selection operating on each site of the ABCB5 sequence. Selecton output showing the level of selection on human ABCB5. Orange residues indicate an ω value greater than 1, suggesting positive selection, while dark purple residues have an ω value significantly lower than 1, indicating strong purifying selection.

A phylogenetic analysis indicates that ABCB5 is most closely related to the other full transporters, ABCB1, ABCB4, and ABCB11 (fig. 5). This clade is supported with a Bayesian posterior probability of 1.0 (i.e., 100%). ABCB1 and 4 are the most closely related, followed by ABCB5, with ABCB11 as the outgroup to these, and with all of these nodes supported by high Bayesian posterior probabilities. The ABCB full transporters originated from a single gene that is present in non-vertebrates and goes back as far as *Drosophila*, *C. elegans*, and yeast. ABCB11 orthologs are present in non-mammals such as *Xenopus*, birds, and reptiles, while the duplication event that led to ABCB1 and ABCB4 occurred after the split of mammals from reptiles and before the most recent common ancestor of extant mammals, i.e., sometime between 310 million years ago [24], [25] and ∼235 million years ago [26]. The only non-mammalian ABCB5 sequence is from *Xenopus*, which suggests the ABCB5 gene may have been lost in some lineages such as birds and reptiles. An analysis of the selection pressure operating on each site reveals that a large proportion of sites in each gene are under strong purifying selection, indicating strong functional constraint (fig. 6).

In phylogenetic analyses the Ka/Ks value is the ratio of the number of nonsynonymous (amino-acid changing) substitutions per nonsynonymous site (Ka) to the number of synonymous (non-amino acid changing) substitutions per synonymous site (Ks). This can be used as an indicator of selective pressure acting on that particular gene. Under neutral conditions Ka/Ks is equal to 1, i.e., mutations occur randomly and the probability of a nonsynonymous mutation becoming fixed in the population is equal to that of a synonymous mutation. A Ka/Ks ratio that is significantly less than 1 indicates that nonsynonymous mutations are less likely to become fixed in a population. This is known as purifying selection as potentially deleterious mutations are purged from the population in order to maintain the sequence of a functionally important site or region within the coding gene.

The REL analysis of DataMonkey found that 628 of the 1,464 codons in the full-length alignment of ABCB5 were highly significant for purifying selection. All of the transmembrane domains and NBDs display low Ka/Ks values, indicating that these domains have been under functional constraint. The predicted first NBD of ABCB5 shows comparable levels of purifying selection to the NBDs of the other ABCB genes, revealing that it has been functionally constrained throughout mammalian evolution, and confirming that ABCB5 has evolved as a full transporter. A comparison between the PAML, DataMonkey, and Selecton results gave no consensus of any sites adjudged to be under strong adaptive selection with high probabilities. However, 2 regions, a cytoplasmic region at the start of the gene and a portion of sites at the end of the first NBD, displayed a number of residues with a Ka/Ks value >1, although these were not found to be significantly greater than 1.

The GABranch analysis of DataMonkey did not suggest that any specific lineages were undergoing a shift to adaptive evolution. The mean Ka/Ks of each branch was estimated to be under 0.5, indicating that ABCB5 has been undergoing strong to moderate purifying selection along each lineage. The probability that any of these branches was undergoing adaptive evolution was estimated at less than 5%. The only exception was the branch leading to the 6 Laurasiatherian taxa (horse, dog, cow, microbat, hedgehog, and shrew), which was estimated to have a Ka/Ks value of 0.75; the probability that this lineage was undergoing positive selection was adjudged to be 33%. The absence of any major shifts in selection between the various lineages would also suggest that the function of ABCB5 has been maintained throughout mammalian evolution.

The Spidermonkey analysis of the full-length *ABCB5* gene returned a pair of residues with a posterior probability of 0.95 of co-evolving. These residues, 444 and 1196 on the human full-length transcript, are in the predicted first and second NBDs, respectively, and thus may be important for interactions between these two domains.

## Discussion

We have shown in this study that according to topological structure, ABCB5.ts (the full transporter) could be a functional form of ABCB5 and demonstrates the conventional 2-TMD and 2-NBD arrangement generally found in full transporters. Each of these TMDs was determined to contain 6 transmembrane helices using HMMTOP software. We also found 2 potential N-glycosylation sites located in the extracellular loops, which may be important for the trafficking of the protein to the plasma membrane. On the other hand, the ABCB5 beta form demonstrates a rather unconventional structure consisting of 2 potential NBDs and 1 potential TMD, suggesting that: (i) this transporter might have a unique catalytic cycle; (ii) some mechanism of post-translational modification may function to potentially edit the beta form into a more conventional structure to give rise to the functional form of the transporter; or finally (iii) this protein may have some function in addition to transport.

Ten non-synonymous cSNPs were found on ABCB5 beta. Of these, one SNP, rs6461515, maps to the potential substrate site (of the protein), suggesting that it may play a role in substrate specificity. We also found this SNP to be slightly more prevalent in South American populations when compared to other populations in the Human Genome Diversity Panel (unpublished data from our laboratory). The rest of these SNPs were in diverse regions of the transporter, including the NBDs. rs58795451, which is in the D-loop of the protein, was determined to be the most deleterious substitution, which agrees well with the fact that the D-loop plays an important role in communication between the NBDs of ABC transporters. The ABCB5.ts (full transporter) has 23 non-synonymous cSNPs, which map to different functional regions of the putative transporter. rs76179099, which maps to the first nucleotide binding domain of the protein, would cause a stop codon to be inserted in this position and may cause the premature translational termination of this ABC protein.

From phylogenetic and evolutionary analyses, we have shown that ABCB5 evolved as a full transporter for most of its evolutionary history. There is an absence of any major shifts in selection between the various lineages, which would suggest that the function of ABCB5 has been maintained throughout mammalian evolution. The full transporter form shows similar patterns of site-specific selection when compared to the other B-family full transporters with high levels of purifying selection in both TMDs and NBDs. This indicates that these motifs have retained functional significance throughout mammalian evolution. The full length predicted form in humans also retains these motifs, suggesting that this could be the functional form with respect to normal physiology. However, this form of the transporter has extremely restricted expression in humans (it has only been documented in the testis) [17] leading to the speculation that ABCB5 beta which has been reported to be the predominant form in melanoma and other cancers may possibly undergo dimerization to create the physiologically relevant transporter in these malignancies. The fact that ABCB5 beta has been implicated in doxorubicin transport in melanoma cells [9] and also in hepatocellular carcinoma cells [13] lends support to this theory.

## Materials and Methods

### Protein Informatics

The HMMTOP program was used to predict the topological structure of ABCB5, using the constraint that the 2^nd^ NBD was located in the cytoplasmic region (www.enzim.hu/hmmtop/). This program operates based on the hypothesis that the topology and localization of the transmembrane segments is determined, not by the specific amino acid composition of the various parts of the protein, but by the amino acid distributions in various regions of the structure [27]. The output of HMMTOP was displayed using the TOPO2 transmembrane protein display software (www.sacs.ucsf.edu/TOPO2/). TMHMM v2 program (www.cbs.dtu.dk/services/TMHMM/) was used to compute the hydrophobicity plots using the hidden Markov model [28]. The functional motifs on the transporter were detected by using the web-based program MOTIFSCAN (http://hits.isb-sib.ch/cgi-bin/PFSCAN), which is primarily a data retrieval and analysis system that can match protein sequences to precomputed patterns and profiles from Pfam and Prosite [29], [30].

Marcoil (www.isrec.isb-sib.ch/webmarcoil/webmarcoilC1.html) [31] and COILS (www.ch.embnet.org/software/COILS_form.html) were used to predict coiled–coil domains. COILS is a program that compares a particular sequence to a database of known parallel two-stranded coiled–coils and computes a similarity score. On comparing this score to the distribution of scores in globular and coiled–coil proteins, the program then calculates the probability that the sequence will adopt a coiled–coil conformation [32].

The Psipred server (http://bioinf.cs.ucl.ac.uk/psipred/) was used to verify that the regions were actually coiled–coils [33]. This program is used to predict protein secondary structure and is based on position-specific scoring matrices created by PSI-BLAST [34].

### Single Nucleotide Polymorphism Analysis

The predicted functional significance of the amino acid substitutions was calculated using Panther software (www.pantherdb.org) and SIFT (http://sift.jcvi.org). Panther estimates the likelihood of a particular nonsynonymous (amino acid-changing) coding SNP to cause a functional impact on the protein. It calculates the subPSEC (substitution position-specific evolutionary conservation) score based on an alignment of evolutionarily related proteins. Then it scores the likelihood of a single amino acid at a particular position (amino acid PSEC/aaPSEC) or the likelihood of the transition of one amino acid to another (substitution PSEC, subPSEC). When aaPSEC  = 0, this is the evolutionarily most common allele (inferred to be definitely functional), whereas more negative values of aaPSEC indicate that the allele is less likely to be observed across evolution (inferred to be less likely to conserve function). The subPSEC score is the difference between the aaPSEC scores for the two alleles. The algorithm takes the absolute value in order to make the scores symmetric, and then multiplies by –1 to adhere to the substitution matrix convention that more negative scores correspond to more severe substitutions. When subPSEC  = 0, the substitution is interpreted as functionally neutral, whereas more negative values of subPSEC predict more deleterious substitutions. The cutoff subPSEC < –3 indicates a deleterious substitution [35]. SIFT uses a sequence homology based approach to classify the substitutions in amino acids. A highly conserved amino acid would be considered to be intolerant to most substitutions while a poorly conserved position would tolerate the majority of substitutions [36].

### Phylogenetic and Evolutionary Analysis

Amino acid and nucleotide sequences of each *ABCB* gene for each species were downloaded from the Ensembl database (http://www.ensembl.org). If more than one transcript was available, the longest transcript was chosen. Alignments were constructed on the amino acid sequences with the online MAFFT server (http://align.bmr.kyushu-u.ac.jp/mafft/online/server/index.html), using the G-INS-i setting [37]. Protein alignments were converted into nucleotide alignments using the online PAL2NAL server (http://www.bork.embl.de/pal2nal/) [38]. A Bayesian phylogeny based on the nucleotide alignment of the complete ABCB family was constructed using MrBayes [39]. The nucleotide model used was a General Time Reversible model with 6 substitution rate parameters and gamma-distributed rate variation with a proportion of invariant sites. The analysis was run for 4 million generations,10 with a sample frequency of 100 and burn-in set to 10,000 (which corresponded to 25% of the sampled trees). Selection analyses were performed on PAML v4.1 [40], DataMonkey (http://www.datamonkey.org/) [41] and Selecton (http://selecton.tau.ac.il/) [42]. Site selection analyses were based on the ratio of the rate of non-synonymous substitutions per non-synonymous site (Ka) to the rate of synonymous substitutions per synonymous site (Ks). This is used as an indicator of the level of selective pressure acting on a protein-coding gene, with values of less than 1 indicative of purifying selection, while values greater than 1 suggest positive selection. Analyses were performed in PAML by comparing a model with one-rate category to a model that added a neutral rate category and then to a 3^rd^ model that added a category for positively selected sites. In DataMonkey, the Random Effects Likelihood (REL) method was used to estimate the Ka/Ks value for each codon.

In Selecton, the mechanistic-empirical combined (MEC) model was used, which takes into account differences between amino-acid replacement probabilities, under the assumption that more radical changes in amino acid type represent a larger evolutionary step. The GABranch algorithm in DataMonkey was used to measure changes in selective pressure acting along different lineages. The Spidermonkey analysis of DataMonkey was used to infer sites with conditional evolutionary dependencies indicating pairs of sites that may have a functional or structural importance.
